# 105 Burning Heart - An Animal Model of Chronic Heart Failure Following Severe Burn Injury

**DOI:** 10.1093/jbcr/irad045.078

**Published:** 2023-05-15

**Authors:** Gabriel Hundeshagen, Viktoria Mertin, Martin Busch, Philipp Thiele, Felix Trogisch, Jörg Heineke, Michael Egger, Hannah Bürkert, Sophie van Linthout, Manuel Mayr, Jens Fielitz, Ulrich Kneser, Patrick Most, Julia Ritterhoff

**Affiliations:** BG Unfallklinik Ludwigshafen, University of Heidelberg, Ludwigshafen, Rheinland-Pfalz; University Hospital Heidelberg, Heidelberg, Baden-Wurttemberg; University Hospital Heidelberg, Heidelberg, Baden-Wurttemberg; University Hospital Heidelberg, Heidelberg, Baden-Wurttemberg; German Center for Cardiovascular Research, Department of Cardiovascular Physiology, Medical Faculty of Mannheim, Mannheim, Baden-Wurttemberg; German Center for Cardiovascular Research, Department of Cardiovascular Physiology, Medical Faculty of Mannheim, Mannheim, Baden-Wurttemberg; University Hospital Heidelberg, Heidelberg, Baden-Wurttemberg; Julius-Maximilians-University Würzburg, Würzburg, Bayern; Berlin Instute of Health at Charite, Experimental Immunocardiology, Berlin, Berlin, Berlin; Cardiovascular Proteomics, King’s College London, London, England; Molecular Cardiology and Muscular Proteostasis, Department of Internal Medicine B, University of Greifswald; German Center for Cardiovascular Research, Greifswald, Mecklenburg-Vorpommern; BG Unfallklinik Ludwigshafen, University of Heidelberg, Ludwigshafen, Rheinland-Pfalz; Molecular and Translational Cardiology Division, University Hospital Heidelberg; 3German Center for Cardiovascular Research, Heidelberg, Baden-Wurttemberg; Molecular and Translational Cardiology Division, University Hospital Heidelberg; 3German Center for Cardiovascular Research, Heidelberg, Baden-Wurttemberg; BG Unfallklinik Ludwigshafen, University of Heidelberg, Ludwigshafen, Rheinland-Pfalz; University Hospital Heidelberg, Heidelberg, Baden-Wurttemberg; University Hospital Heidelberg, Heidelberg, Baden-Wurttemberg; University Hospital Heidelberg, Heidelberg, Baden-Wurttemberg; German Center for Cardiovascular Research, Department of Cardiovascular Physiology, Medical Faculty of Mannheim, Mannheim, Baden-Wurttemberg; German Center for Cardiovascular Research, Department of Cardiovascular Physiology, Medical Faculty of Mannheim, Mannheim, Baden-Wurttemberg; University Hospital Heidelberg, Heidelberg, Baden-Wurttemberg; Julius-Maximilians-University Würzburg, Würzburg, Bayern; Berlin Instute of Health at Charite, Experimental Immunocardiology, Berlin, Berlin, Berlin; Cardiovascular Proteomics, King’s College London, London, England; Molecular Cardiology and Muscular Proteostasis, Department of Internal Medicine B, University of Greifswald; German Center for Cardiovascular Research, Greifswald, Mecklenburg-Vorpommern; BG Unfallklinik Ludwigshafen, University of Heidelberg, Ludwigshafen, Rheinland-Pfalz; Molecular and Translational Cardiology Division, University Hospital Heidelberg; 3German Center for Cardiovascular Research, Heidelberg, Baden-Wurttemberg; Molecular and Translational Cardiology Division, University Hospital Heidelberg; 3German Center for Cardiovascular Research, Heidelberg, Baden-Wurttemberg; BG Unfallklinik Ludwigshafen, University of Heidelberg, Ludwigshafen, Rheinland-Pfalz; University Hospital Heidelberg, Heidelberg, Baden-Wurttemberg; University Hospital Heidelberg, Heidelberg, Baden-Wurttemberg; University Hospital Heidelberg, Heidelberg, Baden-Wurttemberg; German Center for Cardiovascular Research, Department of Cardiovascular Physiology, Medical Faculty of Mannheim, Mannheim, Baden-Wurttemberg; German Center for Cardiovascular Research, Department of Cardiovascular Physiology, Medical Faculty of Mannheim, Mannheim, Baden-Wurttemberg; University Hospital Heidelberg, Heidelberg, Baden-Wurttemberg; Julius-Maximilians-University Würzburg, Würzburg, Bayern; Berlin Instute of Health at Charite, Experimental Immunocardiology, Berlin, Berlin, Berlin; Cardiovascular Proteomics, King’s College London, London, England; Molecular Cardiology and Muscular Proteostasis, Department of Internal Medicine B, University of Greifswald; German Center for Cardiovascular Research, Greifswald, Mecklenburg-Vorpommern; BG Unfallklinik Ludwigshafen, University of Heidelberg, Ludwigshafen, Rheinland-Pfalz; Molecular and Translational Cardiology Division, University Hospital Heidelberg; 3German Center for Cardiovascular Research, Heidelberg, Baden-Wurttemberg; Molecular and Translational Cardiology Division, University Hospital Heidelberg; 3German Center for Cardiovascular Research, Heidelberg, Baden-Wurttemberg; BG Unfallklinik Ludwigshafen, University of Heidelberg, Ludwigshafen, Rheinland-Pfalz; University Hospital Heidelberg, Heidelberg, Baden-Wurttemberg; University Hospital Heidelberg, Heidelberg, Baden-Wurttemberg; University Hospital Heidelberg, Heidelberg, Baden-Wurttemberg; German Center for Cardiovascular Research, Department of Cardiovascular Physiology, Medical Faculty of Mannheim, Mannheim, Baden-Wurttemberg; German Center for Cardiovascular Research, Department of Cardiovascular Physiology, Medical Faculty of Mannheim, Mannheim, Baden-Wurttemberg; University Hospital Heidelberg, Heidelberg, Baden-Wurttemberg; Julius-Maximilians-University Würzburg, Würzburg, Bayern; Berlin Instute of Health at Charite, Experimental Immunocardiology, Berlin, Berlin, Berlin; Cardiovascular Proteomics, King’s College London, London, England; Molecular Cardiology and Muscular Proteostasis, Department of Internal Medicine B, University of Greifswald; German Center for Cardiovascular Research, Greifswald, Mecklenburg-Vorpommern; BG Unfallklinik Ludwigshafen, University of Heidelberg, Ludwigshafen, Rheinland-Pfalz; Molecular and Translational Cardiology Division, University Hospital Heidelberg; 3German Center for Cardiovascular Research, Heidelberg, Baden-Wurttemberg; Molecular and Translational Cardiology Division, University Hospital Heidelberg; 3German Center for Cardiovascular Research, Heidelberg, Baden-Wurttemberg; BG Unfallklinik Ludwigshafen, University of Heidelberg, Ludwigshafen, Rheinland-Pfalz; University Hospital Heidelberg, Heidelberg, Baden-Wurttemberg; University Hospital Heidelberg, Heidelberg, Baden-Wurttemberg; University Hospital Heidelberg, Heidelberg, Baden-Wurttemberg; German Center for Cardiovascular Research, Department of Cardiovascular Physiology, Medical Faculty of Mannheim, Mannheim, Baden-Wurttemberg; German Center for Cardiovascular Research, Department of Cardiovascular Physiology, Medical Faculty of Mannheim, Mannheim, Baden-Wurttemberg; University Hospital Heidelberg, Heidelberg, Baden-Wurttemberg; Julius-Maximilians-University Würzburg, Würzburg, Bayern; Berlin Instute of Health at Charite, Experimental Immunocardiology, Berlin, Berlin, Berlin; Cardiovascular Proteomics, King’s College London, London, England; Molecular Cardiology and Muscular Proteostasis, Department of Internal Medicine B, University of Greifswald; German Center for Cardiovascular Research, Greifswald, Mecklenburg-Vorpommern; BG Unfallklinik Ludwigshafen, University of Heidelberg, Ludwigshafen, Rheinland-Pfalz; Molecular and Translational Cardiology Division, University Hospital Heidelberg; 3German Center for Cardiovascular Research, Heidelberg, Baden-Wurttemberg; Molecular and Translational Cardiology Division, University Hospital Heidelberg; 3German Center for Cardiovascular Research, Heidelberg, Baden-Wurttemberg; BG Unfallklinik Ludwigshafen, University of Heidelberg, Ludwigshafen, Rheinland-Pfalz; University Hospital Heidelberg, Heidelberg, Baden-Wurttemberg; University Hospital Heidelberg, Heidelberg, Baden-Wurttemberg; University Hospital Heidelberg, Heidelberg, Baden-Wurttemberg; German Center for Cardiovascular Research, Department of Cardiovascular Physiology, Medical Faculty of Mannheim, Mannheim, Baden-Wurttemberg; German Center for Cardiovascular Research, Department of Cardiovascular Physiology, Medical Faculty of Mannheim, Mannheim, Baden-Wurttemberg; University Hospital Heidelberg, Heidelberg, Baden-Wurttemberg; Julius-Maximilians-University Würzburg, Würzburg, Bayern; Berlin Instute of Health at Charite, Experimental Immunocardiology, Berlin, Berlin, Berlin; Cardiovascular Proteomics, King’s College London, London, England; Molecular Cardiology and Muscular Proteostasis, Department of Internal Medicine B, University of Greifswald; German Center for Cardiovascular Research, Greifswald, Mecklenburg-Vorpommern; BG Unfallklinik Ludwigshafen, University of Heidelberg, Ludwigshafen, Rheinland-Pfalz; Molecular and Translational Cardiology Division, University Hospital Heidelberg; 3German Center for Cardiovascular Research, Heidelberg, Baden-Wurttemberg; Molecular and Translational Cardiology Division, University Hospital Heidelberg; 3German Center for Cardiovascular Research, Heidelberg, Baden-Wurttemberg; BG Unfallklinik Ludwigshafen, University of Heidelberg, Ludwigshafen, Rheinland-Pfalz; University Hospital Heidelberg, Heidelberg, Baden-Wurttemberg; University Hospital Heidelberg, Heidelberg, Baden-Wurttemberg; University Hospital Heidelberg, Heidelberg, Baden-Wurttemberg; German Center for Cardiovascular Research, Department of Cardiovascular Physiology, Medical Faculty of Mannheim, Mannheim, Baden-Wurttemberg; German Center for Cardiovascular Research, Department of Cardiovascular Physiology, Medical Faculty of Mannheim, Mannheim, Baden-Wurttemberg; University Hospital Heidelberg, Heidelberg, Baden-Wurttemberg; Julius-Maximilians-University Würzburg, Würzburg, Bayern; Berlin Instute of Health at Charite, Experimental Immunocardiology, Berlin, Berlin, Berlin; Cardiovascular Proteomics, King’s College London, London, England; Molecular Cardiology and Muscular Proteostasis, Department of Internal Medicine B, University of Greifswald; German Center for Cardiovascular Research, Greifswald, Mecklenburg-Vorpommern; BG Unfallklinik Ludwigshafen, University of Heidelberg, Ludwigshafen, Rheinland-Pfalz; Molecular and Translational Cardiology Division, University Hospital Heidelberg; 3German Center for Cardiovascular Research, Heidelberg, Baden-Wurttemberg; Molecular and Translational Cardiology Division, University Hospital Heidelberg; 3German Center for Cardiovascular Research, Heidelberg, Baden-Wurttemberg; BG Unfallklinik Ludwigshafen, University of Heidelberg, Ludwigshafen, Rheinland-Pfalz; University Hospital Heidelberg, Heidelberg, Baden-Wurttemberg; University Hospital Heidelberg, Heidelberg, Baden-Wurttemberg; University Hospital Heidelberg, Heidelberg, Baden-Wurttemberg; German Center for Cardiovascular Research, Department of Cardiovascular Physiology, Medical Faculty of Mannheim, Mannheim, Baden-Wurttemberg; German Center for Cardiovascular Research, Department of Cardiovascular Physiology, Medical Faculty of Mannheim, Mannheim, Baden-Wurttemberg; University Hospital Heidelberg, Heidelberg, Baden-Wurttemberg; Julius-Maximilians-University Würzburg, Würzburg, Bayern; Berlin Instute of Health at Charite, Experimental Immunocardiology, Berlin, Berlin, Berlin; Cardiovascular Proteomics, King’s College London, London, England; Molecular Cardiology and Muscular Proteostasis, Department of Internal Medicine B, University of Greifswald; German Center for Cardiovascular Research, Greifswald, Mecklenburg-Vorpommern; BG Unfallklinik Ludwigshafen, University of Heidelberg, Ludwigshafen, Rheinland-Pfalz; Molecular and Translational Cardiology Division, University Hospital Heidelberg; 3German Center for Cardiovascular Research, Heidelberg, Baden-Wurttemberg; Molecular and Translational Cardiology Division, University Hospital Heidelberg; 3German Center for Cardiovascular Research, Heidelberg, Baden-Wurttemberg; BG Unfallklinik Ludwigshafen, University of Heidelberg, Ludwigshafen, Rheinland-Pfalz; University Hospital Heidelberg, Heidelberg, Baden-Wurttemberg; University Hospital Heidelberg, Heidelberg, Baden-Wurttemberg; University Hospital Heidelberg, Heidelberg, Baden-Wurttemberg; German Center for Cardiovascular Research, Department of Cardiovascular Physiology, Medical Faculty of Mannheim, Mannheim, Baden-Wurttemberg; German Center for Cardiovascular Research, Department of Cardiovascular Physiology, Medical Faculty of Mannheim, Mannheim, Baden-Wurttemberg; University Hospital Heidelberg, Heidelberg, Baden-Wurttemberg; Julius-Maximilians-University Würzburg, Würzburg, Bayern; Berlin Instute of Health at Charite, Experimental Immunocardiology, Berlin, Berlin, Berlin; Cardiovascular Proteomics, King’s College London, London, England; Molecular Cardiology and Muscular Proteostasis, Department of Internal Medicine B, University of Greifswald; German Center for Cardiovascular Research, Greifswald, Mecklenburg-Vorpommern; BG Unfallklinik Ludwigshafen, University of Heidelberg, Ludwigshafen, Rheinland-Pfalz; Molecular and Translational Cardiology Division, University Hospital Heidelberg; 3German Center for Cardiovascular Research, Heidelberg, Baden-Wurttemberg; Molecular and Translational Cardiology Division, University Hospital Heidelberg; 3German Center for Cardiovascular Research, Heidelberg, Baden-Wurttemberg; BG Unfallklinik Ludwigshafen, University of Heidelberg, Ludwigshafen, Rheinland-Pfalz; University Hospital Heidelberg, Heidelberg, Baden-Wurttemberg; University Hospital Heidelberg, Heidelberg, Baden-Wurttemberg; University Hospital Heidelberg, Heidelberg, Baden-Wurttemberg; German Center for Cardiovascular Research, Department of Cardiovascular Physiology, Medical Faculty of Mannheim, Mannheim, Baden-Wurttemberg; German Center for Cardiovascular Research, Department of Cardiovascular Physiology, Medical Faculty of Mannheim, Mannheim, Baden-Wurttemberg; University Hospital Heidelberg, Heidelberg, Baden-Wurttemberg; Julius-Maximilians-University Würzburg, Würzburg, Bayern; Berlin Instute of Health at Charite, Experimental Immunocardiology, Berlin, Berlin, Berlin; Cardiovascular Proteomics, King’s College London, London, England; Molecular Cardiology and Muscular Proteostasis, Department of Internal Medicine B, University of Greifswald; German Center for Cardiovascular Research, Greifswald, Mecklenburg-Vorpommern; BG Unfallklinik Ludwigshafen, University of Heidelberg, Ludwigshafen, Rheinland-Pfalz; Molecular and Translational Cardiology Division, University Hospital Heidelberg; 3German Center for Cardiovascular Research, Heidelberg, Baden-Wurttemberg; Molecular and Translational Cardiology Division, University Hospital Heidelberg; 3German Center for Cardiovascular Research, Heidelberg, Baden-Wurttemberg

## Abstract

**Introduction:**

Our clinical research unveiled chronic heart failure with preserved ejection fraction (HFpEF) as a long-term sequel in survivors of severe pediatric burn injury due to a yet unknown molecular pathomechanism. Applying a standardized rat model, we systematically determined the pathophysiological impact of burn injury on long-term cardiac performance to uncover systemic and molecular pathomechanisms that may cause post-burn HFpEF development.

**Methods:**

Male adolescent SD-rats were subjected to a 60 % total body surface area (TBSA) full-thickness burn- or sham-trauma and subsequently characterized after burn-injury by serial transthoracic echocardiography, bulk myocardial next-generation sequencing and proteomics as well as RT-PCR, immuno-blotting (IB), histology and plasma proteomics for cardiac performance and molecular alterations, respectively, at 3, 7, 30 and 90days.

**Results:**

In comparison to the sham-group (SG), animals from the burn-group (BG) recapitulated typical post-burn clinical traits, such as significant loss in body weight (BG 27 % less than SG at 30d, p< 0.05) or skeletal muscle wasting (27 % less at 30d, p< 0.05) in accord with elevated molecular atrophy markers. We show post-burn cardiac muscle wasting (BG 22 % less at 30d, p< 0.05) and persistent markers of cardiac dysfunction in accord with significant histological cardiomyocyte hypotrophy (BG -8 % at 30d, p< 0.05) and significantly diminished left ventricular (LV) global longitudinal strain and isovolumic relaxation time in BGs, while LV-EF remained unchanged. Weighted gene network correlation analysis from bulk myocardial NGS and clinical traits related activation of immunological and pro-fibrotic pathways in post-burn injury hearts to cardiac dysfunction in BGs. Subsequent RT-PCR and histology confirmed significant myocardial accumulation of cardio-depressive damage associated molecular patterns (i.e., S100A8 and A9) and infiltration by granulocytes and monocytes as well as significant LV fibrosis. Serial plasma proteomic analysis indicated elevated plasma levels of S100A8 and A9 and other heart failure markers that mirrored similar changes in human post-burn plasma samples.

**Conclusions:**

Here we report the development of HFpEF as a novel systemic consequence of severe burn injury in a rodent model, which warrants further mechanistic and translational studies. Cardiac inflammation and fibrosis are known to negatively impact cardiac performance and may be mechanistic key findings that will guide further therapeutic studies and subsequent validation of post-burn heart failure biomarkers

**Applicability of Research to Practice:**

This model is part of a translational and interdisciplinary experimental and clinical effort to inform pathophysiology and mechanistics of long-term heart failure in burn patients. Echocardiographic data and parameters from this model are currently being used to evaluate adult survivors of severe burn injury for signs of HFpEF.

